# Osteoproductivity in Experimentally Induced Cranial Bone Defects in Rabbits

**DOI:** 10.61186/ibj.3940

**Published:** 2023-09-09

**Authors:** Alkın Ünsal, Ercan Durmuş, İlhami Çelik

**Affiliations:** Selçuk University, Faculty of Dentistry, Department of Oral and Maxillofacial Surgery, Kampüs 42080, Konya, Türkiye

**Keywords:** Carboxymethyl cellulose, Eggshell particles, Gelatin

## Abstract

**Background:**

Autogenous bone grafts are the gold standard for being used as graft materials in maxillofacial surgery. However, a limited amount of these materials is available from the donor site, and there is also more need for a larger operating area and a second surgery, which frequently leads to unreliable graft incorporation, tooth ankylosis, and root resorption. Therefore, newer bone graft substitutes have been developed as alternatives, among which eggshell powder has been introduced as a bone substitute. This study aimed to evaluate the biocompatibility, resorption kinetics, and osteoproductivity of the unprocessed, CMC-coated, and gelatin-coated ostrich eggshell particles.

**Methods:**

Four half-thickness calvarial defects were created in each animal. At the end of the 1^st^ and 3^rd^ months, the defected sites were investigated by clinical, histological, radiological and histomorphometrical methods.

**Results:**

Coating the eggshell particles with CMC and gelatin facilitated their surgical application and contributed to new bone formation. However, their newly formed bone rate at the 3^rd^ month was lower than those of the unprocessed eggshell particles. The CMC coating was more effective than gelatin coating in the bone modeling process.

**Conclusion:**

Ostrich eggshell particles either in native form or coated with CMC could be used as a bone filler for supporting new bone formation and healing in treatment of osseous defects.

## INTRODUCTION

In the reconstruction of cranial, craniofacial, and oral defects, complication-free and uneventful healing at the defect site is of utmost importance^[^^1^^]^. Using a bone graft seems necessary for complete healing, and new bone formation is expected in the defect site. Bone graft materials used as space fillers, should be osteoinductive or osteoconductive and are able to bear the exposed forces, as well as easily gain the desired shape and size. Although autogenous bone grafts are the gold standard graft materials in maxillofacial surgery, intraoral donor sites have limited the availability, and a wider or second surgery is essential. Besides it, unreliable graft incorporation, tooth ankylosis, and root resorption are often observed^[^^1^^-^^3^^]^. Therefore, a variety of bone graft substitutes have been developed as alternatives to autogenous grafts^[^^4^^]^. Ostrich eggshell powder is one of these alternatives introduced as a space-filling bone substitute^[^^5^^]^.

The ostrich eggshell alone or in combination with eggshell membranes have been experimented as a candidate bone substitute^[^^1^^,^^3^^]^. Bone grafts of ostrich eggshell origin are preferable because of their physical and mechanical features and noncollagenous bone matrix protein content^[^^1^^,^^3^^,^^6^^]^. Previous studies have suggested that particulate form of the ostrich eggshell is a resorbable osteoproductive material^[^^3^^,^^7^^]^. 

A graft particle should have a critical size. The kinetic degradation capacity and resorption kinetics of the ostrich eggshell particles are mainly dependent on the size of the particles^[^^1^^,^^3^^,^^8^^,^^9^^]^. The larger particles, which have a relatively lower resorption rate, seem to have a more osteogenic activity^[^^1^^,^^3^^]^. A bioactive material have to be slowly dissolved in the defect site by forming a layer of biological apatite and form a direct bond to the osseous tissue^[^^9^^]^. Moreover, a resorbable material allows infiltration of a new tissue inside its irregular cavities, without interacting with the bone. Although collagen is used in clinical medicine^[^^10^^]^, some deficiencies limit its widespread use. Therefore, gelatin derived from collagen has been preferred as a biomaterial in tissue engineering and new bone formation has been evidenced in the gelatin sponge^[^^10^^,^^11^^-^^15^^]^. 

CMC is a safe and inert biopolymer^[^^16^^]^, which its binding and thickening potential is beneficial for enhancing the clinical characteristics of particulate bone grafts. CMC has been shown to elevate the handling properties of particulate composites of calcium sulfate-demineralized bone matrix^[^^16^^,^^17^^]^. Although CMC is well tolerated and it possesses osteoconductive properties, it may obstruct bone formation in gel form^[^^17^^-^^19^^]^. Despite favorable clinical outcomes, there is limited histological evidence about the effect of CMC on bone regeneration.

The rabbit calvarial defect model has a high priority in the experimental graft studies^[^^1^^,^^4^^]^. The size of bone defect and distances between the defects are of great importance. Bone defects smaller than a certain size(CSD) heal with no damage, whereas a bone defect larger than the CSD remains as a shallow depression in its place^[^^20^^]^. In rabbits, a 6-mm defect in the calvarium is considered a CSD^[^^2^^]^.

Although defects with close proximity can be created in a relatively narrow area of the cranial bones, uneventful healings can occur in these defects. Of note, no interference has been reported among the defect sites treated with different materials^[^^19^^,^^20^^]^.

In this experimental study, we evaluated the biocompatibility, resorption, and osteoproductivity of the unprocessed, CMC-coated or gelatin-coated graft substitutes derived from ostrich eggshells by clinical, radiological, histological, and histomorphometrical methods in CSDs in rabbit calvaria.

## MATERIALS AND METHODS

**Animals and experimental groups**

In this study, 20 mature male White New Zealand rabbits aged 10-12 months, weighing 3.3-4.2 kg (mean 3.7 kg ± 0.15), were used. To avoid individual differences, we created four defects in the skull of each animal; one of them was left empty as a control group, while the others were filled with 75 mg of egg shell powder particles processed by different methods. 

**Preparation of the graft material**

Fresh ostrich (Struthio camelus) eggshell pieces were washed with double distilled water, powdered and then treated with 2.5% glutaraldehyde (Merck, Darmstadt, Germany) for 24 hours. The ostrich eggshell particles of 250-500 µm were collected and rinsed three times. Half of the particles were coated with gelatin as described before^[^^20^^]^. Coating by CMC was accomplished by immersing the eggshell particles in 5% of CMC solution under 300 mbar vacuum overnight. The particles were then sterilized with ethylene oxide (AX-400, Axis®, İzmir, Türkiye) and kept at 4 °C until use. 

**Surgical procedures**

**Fig. 1:**
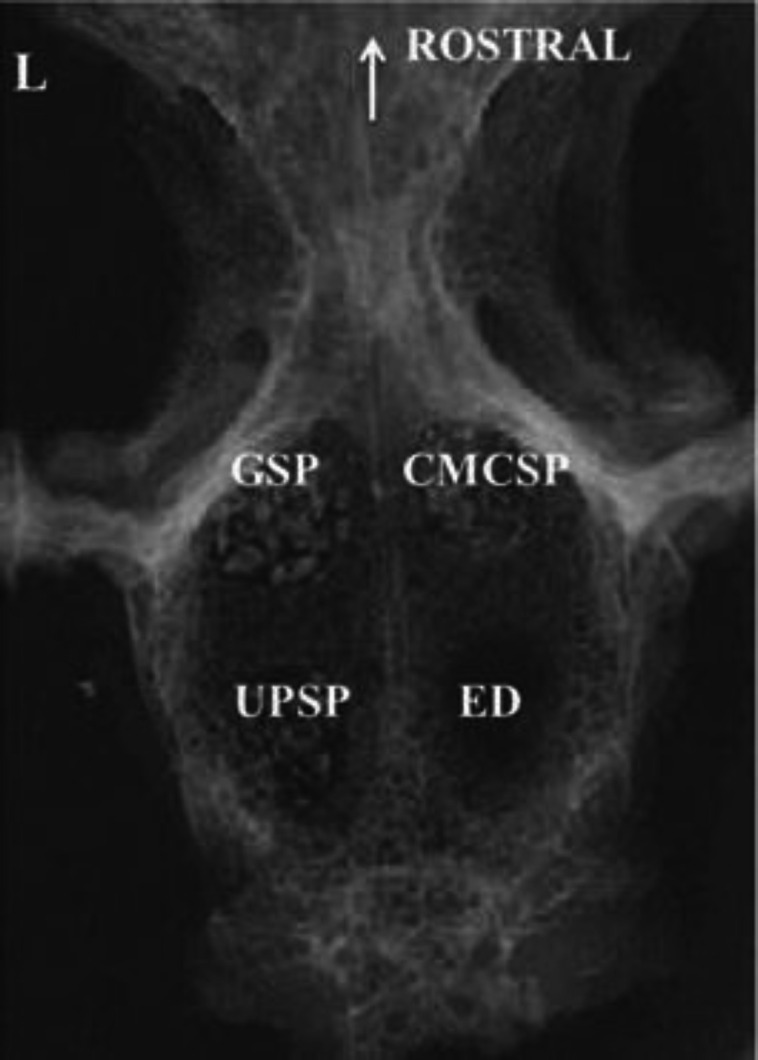
Post-operative dorsoventral radiographic view of the operated region. Arrow indicates rostral direction

All the surgical procedures were performed under general anesthesia induced by 20 mg/kg xylazine (RompunR Bayer; Leverkusen, Germany) and 10 mg/kg ketamine hydrochloride (KetanesR; Alke, Türkiye). A midline skin incision was made along the midsagittal suture of the skull, and then both the skin and underlying tissues were deviated bilaterally to expose the entire calvarium. In the outer cortical half of each calvarium, two defects were created on both sides, i.e. a total of four defects. The right caudal defect remained empty (ED group) and served as the control group. The right cranial defects were filled with CMC-coated ostrich eggshell particles (CMCSP group). The left cranial and the left caudal defects were filled with gelatin-coated ostrich eggshell particles (GSP group), and the native eggshell particles (UPSP group), respectively. The defect sites were closed with two layers of interrupted sutures. The animals were then received intramuscular injections, 0.5 mL/kg of antibiotics (Penicillin G, Procaine 400,000 IU/mL, Fako®, Istanbul, Türkiey). Following each surgical operation, dorsoventral radiographic images of the cranium were taken (Fig. 1).  At the end of the 1^st ^and 3^rd^ months after surgery, 10 animals were sacrificed by intraperitoneal injection of an overdose of pentobarbital (100 mg/kg; Nembutal®, 100 mg/mL, Abbott Laboratories, Chicago, IL, USA).

**Radiography**

Graft-implanted calvarial regions were dissected out, dorsoventral radiographic images were taken and then the samples were immersed in phosphate-buffered saline (0.1 M; pH 7.4). 

**Histology and histomorphometry**

Calvaria samples were first fixed in formalin (10%) prior to decalcification in ethylene diamine tetra acetic acid (10%), processed, and then immersed in paraffine blocks. The 5-µm thick sections of calvaria were stained using hematoxylin-eosin, Crossmon’s trichrome, and Schmorl's picro-thionin stains. The stained sections were examined under a light microscope (Nikon Eclipse E 400, Nikon Corporation, Chiyoda-ku, Japan), and digital images were recorded. The contour of the periosteum, integration and resorption degree of the particles, foreign body reaction, newly formed bone tissue, and osteoblastic and osteoclastic activities in the defected sites were evaluated. Also, the mean percentages of the graft particles, connective tissue, and newly formed bone tissue in the defected sites were determined using BS 200 PRO^®^ image analysis software (BAB Soft Engineering, Medical Industry and Trade, Ankara, Türkiye).  

**Statistical analysis**

Normal distribution of the data was confirmed by the Kolmogorov-Smirnov test. The significance of the differences among the groups was determined using the one-way variance analysis (ANOVA), as well as post hoc and HSD tests. The values (p < 0.05) were regarded as statistically significant.

## RESULTS

**Clinical findings**

No complications due to the surgical procedures developed throughout the study. No discharge or clinical signs of inflammation were also observed. 

**Radiological and CT findings**

**Fig. 2:**
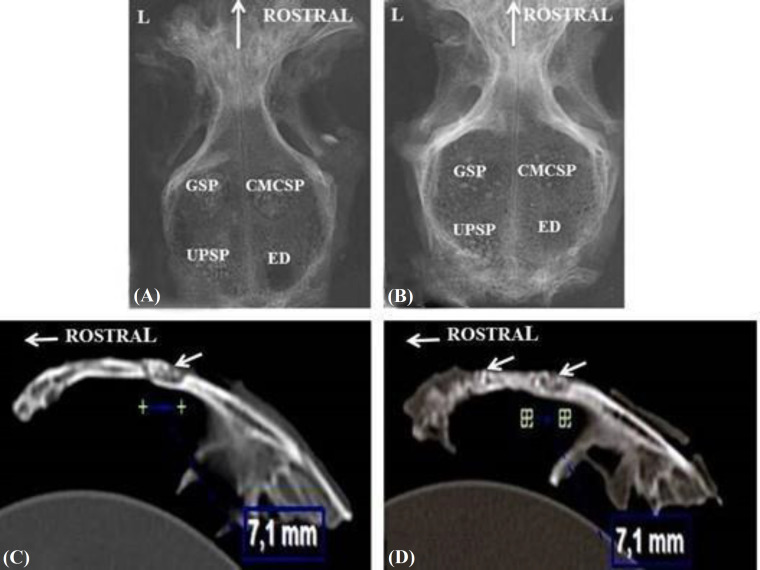
Radiographic views of the defects sites at the end of the (A) 1^st^ and (B) 3^rd^ months after the surgery. Vertical arrows indicate rostral direction. (C) A CT image of the UPSP-filled defect at the end of the 1^st^ month; horizontal arrow indicates rostral direction and the oblique arrow indicates the defect site. (D) A CT image of the GSP (the left oblique arrow) and CMCSP-filled (the right oblique arrow) defects at the end of the 3^rd^ month after the surgery; horizontal arrow indicates rostral direction. Symbols on Figures 2C and 2D show the areas measured

At the end of the postoperative 1^st^ and 3^rd^ months, the defected sites were clearly differentiated from the surrounding tissues as partial radiolucent regions. Although the opacity of the graft particles was lower at the end of the 3^rd^ month, the particles were recognized in both time periods (Fig. 2).  

**H** **istologic findings**

**Fig. 3.**
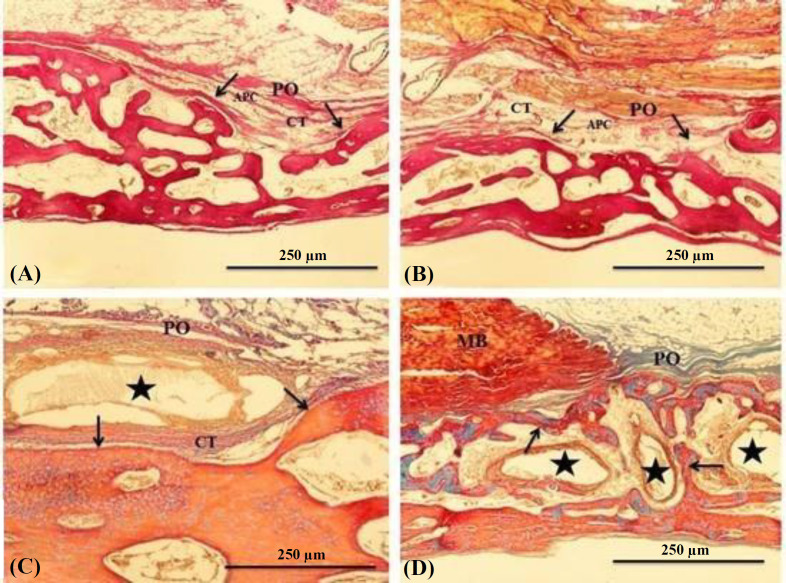
(A) A section of the ED group at the end of the 1^st^ month. Central depression of PO and narrow rim of the new formed osseous tissue lining defected margin (arrows) are seen. (B) A section of the ED group at the end of the 3^rd^ month. The defect site has quite similar histology with 1^st^ month. (C) A section of the UPSP defect at the end of 1^st^ month. (D) A section of the UPSP defect at the end of 3^rd^ month. PO, periosteum; arrows, newly formed bone trabecules; asterisks, graft particles; MB, muscle bundles; CT, connective tissue; APC, adipocytes

**Fig. 4:**
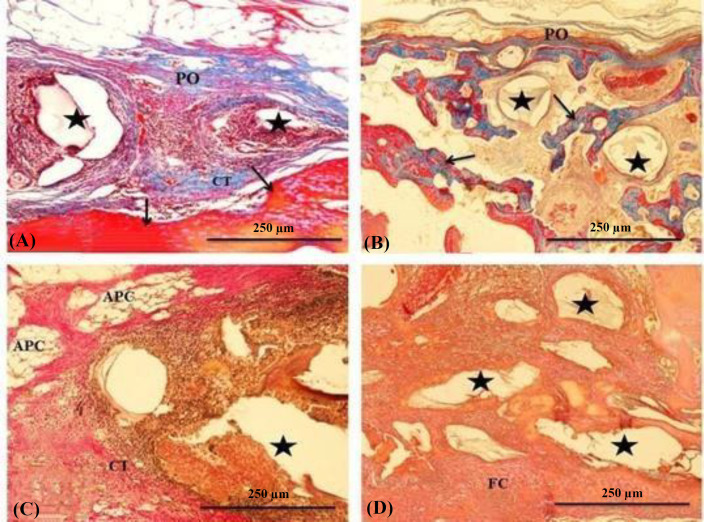
(A) A section of the CMCSP defected site at the end of 1^st^ month. The area is covered with an undepressed continuous PO. Asterisks show advancedly resorbed graft particles, and arrows indicate newly formed bone. (B) A section of the CMCSP defect at end of the 3^rd^ month. An undepressed and continuous periosteum (PO) covers the defect surface. Asterisks show graft particles, and arrows indicate bone trabeculae. (C and D). Sections of the GSP defected site at the end of (C) 1^st^ and (D) 3^rd^ months. Asterisks show graft particles. CT, connective tissue; APC, adipocytes; FC, fibrocartilage tissue

In the ED group, the periosteum was continuous and depressed by the end of the 1^st^ month. Severe inflammatory changes were not observed in the defected site; however, it was filled with the vascular, loose connective tissues containing adipocytes. Newly formed bone with definite osteoblastic activity lined the defected margins (Fig. 3A). At the end of the 3^rd^ month, in addition to the enlargement of the newly formed bone, defected sites displayed similar histology to the 1^st^ month (Fig. 3B). In the UPSP group, the defected surface was covered with unpitted periosteum at the end of the 1^st ^month, and the defected margin was limited by the newly formed osseous tissue. Large particles were partially absorbed, but the small particles were fully absorbed. Connective tissue trabeculae rich in blood vessels filled the areas between the graft particles. Although weak mononuclear cell infiltration was observed in the connective tissue in a few defect areas, foreign body giant cells were not detected in any sample. Newly formed osseous tissue was not observed either on the particle surfaces or between the particles (Fig. 3C). At the end of the 3^rd^ month, a continuous and undepressed periosteum covered the defect, and the smaller particles were highly absorbed. The defect margin and intertrabecular regions were filled with the newly formed trabecular bone tissue (Fig. 3D). In the CMCSP group, all the defected sites were covered with a continuous and undepressed periosteum at the end of the 1^st^ month. Newly formed osseous tissue occupied the defect margin, and the graft particles were resorbed at different levels in the defected sites. Some of the particles were surrounded by a fibrous connective tissue layer. Foreign body giant cells were not observed in the defected sites. Newly formed bone was observed neither on the particle surfaces nor in the interparticle regions. (Fig. 4A). At the end of the 3^rd^ month, primary bone trabeculae occupied the space among the particles. A thin connective tissue rim covered some of the highly resorbed graft particles (Fig. 4B).  In the GSP group, 

the periosteum was a continuous fibrous layer. Periosteal depression was not observed, and the resorption of the grafted particles in the defected sites was in an advanced stage. A vascular and loose connective tissue occupied the interparticle region of the defect. Mononuclear cells were heavily infiltrated into the connective tissue surrounding the graft particles; however, foreign body giant cells were not found (Fig. 4C). At the end of the 3^rd^ month, the interparticle region was primarily occupied with fibrous connective tissue and fibrocartilage tissue trabeculae. Newly formed osseous tissue was mainly identified in the defect margin (Fig. 4D).

**Histomorphometrical results**

The histomorphometrical results of the groups are shown in Table 1. At the end of the 1^st ^month, the particle and connective tissue percentages of the experimental groups were high, which significantly decreased at the end of the 3^rd^ month (p < 0.05). Graft particle rates were quite similar at all time periods of the experiment (p > 0.05). The newly formed bone tissue rates were significantly higher at the end of the 3^rd^ than that of 1^st^ month (p < 0.05). The CMSP and GSP groups displayed significantly higher osseous tissue rates than the ED group (p < 0.05). The highest bone tissue rate was observed in the UPSP group, followed by the CMCSP group. However, the bone rate of the GSP group was significantly lower than that of the UPSP and CMCSP groups (p < 0.05).

## DISCUSSION

Most maxillofacial bone defects in the CSD range are healed through the self-repairing capacity of the bone tissue, and those larger than CSD do not heal completely. Scientists and surgeons have long been developed as alternatives to autogenous grafts^[^^4^^]^. Ostrich eggshell powder is one of these alternatives introduced as a space-filling bone substitute^[^^5^^]^. However, it is of great importance to understand the bioactivity and resorption kinetics of bone substitutes in experimental models before implementing in the clinic. In this study, the bilateral rabbit calvarial defect model was chosen because it is not prone to spontaneous healing due to poor blood flow and membranous origin of the bones^[^^5^^]^, as well as the risk of damage to the superior sagittal sinus is low, and the surgical operation can be easily performed^[^^1^^,^^3^^]^.

The size of defect is an important criterion that substantially affects defect healing. An osseous defect that does not heal spontaneously is regarded as a CSD. Different CSDs in the range of 10-15 mm have been considered to be suitable^[^^1^^,^^3^^]^. In this study, a model of 6-mm half-thickness calvarial defect was selected. Given that substances may leak from one defect area, which affects the healing events in the other defect areas^[^^1^^,^^3^^]^, four defects were created in each animal in order to ensure safe distances between defect areas.

 Although most of the autografts and allografts met most of the requirements expected from a reasonable graft^[^^21^^]^, limited amount of the grafts are available and a large amount of donor tissues are damaged. Besides, there are difficulties in obtaining graft materials in the desired shape^[^^22^^]^. Many candidate materials with different origins have been introduced as bone substitutents^[^^23^^,^^24^^]^. As the chemical content of the eggshell is quite similar to that of the bone, and its biodegradation, biocompatibility, attachment to the original tissue of the recipient, and osteoproductivity are at reasonable levels, an eggshell graft is mainly regarded a candidate for bone substitute in dentistry^[^^1^^,^^3^^,^^7^^-^^9^^]^. Egg shell contains many bioactive molecules, some of which play an active role in the biomineralization and control of this process during construction of the shell^[^^6^^]^. Ostrich egg is a preferable graft material source because of its size, endurance, curvature, and perfect mechanical features^[^^1^^,^^3^^]^.

Eggshell particles used as bone implants are absorbed in two critical stages: dissolution and subsequent engulfment by macrophages without causing immunogenic reactions. In this study, ostrich eggshell particles (250-500 µm) were used as a graft material in the native CMC-adsorbed, and gelatin-adsorbed forms, since the osteoproductive activity of these particles has been reported by previous researchers^[^^1^^,^^3^^]^. Moreover, eggshell contains many bioactive molecules, such as osteopontin, a proteoglycan plays significant roles in calcification via enhancing the adhesion of osteoblasts to matrix and binding to hydroxyapatite^[^^1^^,^^3^^]^.

At the end of the 1^st^ month of the experiment, the defect site of the ED group and the space between the shell particles in the experimental groups were filled with a well-vascularized fibrous connective tissue rich in fibroblasts^[^^2^^,^^24^^,^^25^^]^. Newly formed bone was confined to the defect margins in all defect areas by the end of the 1^st^ month, as shown in a previous study^[^^26^^]^. The UPSP, CMCSP, and GSP groups had a greater rate of the newly formed bone compared with ED defects in the healing periods, i.e. the 1^st^ and 3^rd ^months. Our results indicate that in the native and coated eggshell particle-filled defects, a favorable amount of the new bone forms and progressive replacement of the grafted particles with newly formed bone takes place with increasing healing time. However, coating the eggshell particles either with CMC or gelatin did not exert a striking effect on osteoproduction. This result may be due to the barrier effect of CMC and gelatin, which prevent the direct contact of host cells with eggshell particles and thus inhibits the diffusion of substances released from these particles into the tissue fluid. Developing the tissue responses by the host against the implanted biomaterials are mainly determined using the morphology, composition, and implanted area of the biomaterial^[^^27^^,^^28^^]^. Mild inflammatory reactions are observed around ostrich eggshell particles at the end of the 1^st^ month^[^^2^^,^^5^^]^. Severe foreign body rejection reaction has not been observed at the end of the 1^st^ or 3^rd^ month. Nevertheless, a mild mononuclear cell infiltration was prominent in the GSP group throughout the experiment, which may be due to the mild antigenicity of gelatin. 

**Table 1:** Histomorphometrical results of the groups at the end of the 1^st ^and 3^rd ^months (mean ± SD)

**Time**	**Groups**	**Graft particle area (%)**	**Connective tissue area (%)**	**Newly formed bone area (%)**
1^st^ month	ED	nd	93.60 ± 0.4^a^	6.40 ± 0.4^b^
	UPSP	45.16 ± 0.3	38.70 ± 0.4^c^	16.14 ± 0.3^a^
	CMCSP	44.47 ± 0.2	39.77 ± 0.2^b^	15.76 ± 0.4^a^
	GSP	44.22 ± 0.3	40.30 ± 0.4^b^	15.48 ± 0.3^a^
				
3^rd^ month	ED	nd	85.88 ± 0.4^a^	14.12 ± 0.2^d^
	UPSP	38.01 ± 0.5^a^	25.31 ± 0.4^d^	36.68 ± 0.4^a^
	CMCSP	39.07 ± 0.4^a^	27.40 ± 0.2^c^	33.53 ± 0.3^b^
	GSP	38.15 ± 0.2^a^	38.68 ± 0.4^b^	33.17 ± 0.4^c^

^a-d^The differences among the values with different superscripts in a column are statistically significant (p < 0.05); nd, not determined

Size of the exposed surface of a defect is important, since a greater area allows a greater number of osteoinductive factors to be available for osteogenesis. The microporous surface structure of bone substitutes contributes to the increased new bone formation, via enhancing osteogenesis^[^^28^^,^^29^^]^. Because a controlled solubility rate is required to help bone formation, particle size has a vital role in osteogenic capability. Powdered material provides the maximum interaction surface with the recipient’s target cells and thus might show suitable osteogenic activity; however, smaller particles are resorbed quickly. Nevertheless, small particles dissolve rapidly and the defected site, may be sooner than the time that they require to remain in the site. This occurrence results in the lack of osteogenic stimuli and excessive foreign body reactions. Initial pH, ionic concentration, and temperature are significant factors in degradation and dissolution of the graft particles. Change in pH affects the rate of dissolution, and pH level above the critical value leads to cytotoxicity^[^^30^^]^. For this reason, the particle size and dissolution rate of the material have to be controlled. Low-soluble materials are used if the material had a long life. Thus, slow-resorbing bone substitutes are advantageous because they can maintain the augmented tissue volume for a longer time^[^^30^^]^. Since high-rate or extremely slow-rate graft resorption can inhibit bone healing, bone grafts that are gradually resorbed and replaced with newly formed bone tissue provide more harmonious new bone formation and bone remodeling^[^^7^^,^^31^^]^. In the present study, 250-500 µm particles were used in which total resorption of the larger particles was not observed over a three-month period. This result indicates that eggshell particles remain in the defected area as long as they are needed during the healing process.

In our study, we preserved the bioactive proteins that both play a crucial role in crystal growth during eggshell formation and were effective in the onset of osteogenic activity of the particles by treating them with 2.5% glutaraldehyde and sterilizing them with ethylene oxide. Moreover, a bone substitute has be easily shaped, handled and able to maintain its initial volume^[^^32^^]^. In the present study, CMC and gelatin coating facilitated grafting of particles with no adverse effects. Similarly, there were no significant side effects in soft tissues injected with chemically cross-linked, pharmacological-grade sodium CMC in previous studies^[^^31^^,^^32^^]^. Although gelatin is considered as a suitable biomaterial to mimic the extracellular matrix^[^^33^^]^, it has low stability in the physiological conditions; therefore, it is recommended to use gelatin with other substances. Demineralized bone matrix gelatin scaffolds have been suggested to be used in bone reconstruction^[^^34^^]^. 

The results of this study showed that in the control group, at the end of the 3^rd ^month, the defective area continued to exist as a shallow cavity and new bone formation was specific to the base and lateral borders of the defect area. In the experimental groups, the defective area was filled with newly formed bone trabeculae and the graft particles absorbed at different levels. No tissue rejection reaction occurred against the graft particles prepared from ostrich egg shell. While the newly formed bone tissues were similar in the experimental groups at the end of the 1^st^ month after surgery, they were lower in the GSP group than the UPSP and CMCSP groups at the end of the 3^rd^ month. This result may be due to the early degradation and resorption of the eggshell particles in the GSP group.

Based on the findings of this study, it can be concluded that ostrich egg shell in its natural form or coated with CMC or gelatin can serve as bone-forming defect filler in the treatment of bone defects. The coating provided ease of application for the graft material during surgery. CMC coating increases new bone formation more than gelatin coating. However, more detailed studies with long-term evaluations are needed to support these results.

## DECLARATIONS

### Ethical statement

All animal care and study protocols were approved by the Institutional Experimental Animal Care and Use Committee of Necmettin Erbakan University, Konya, Türkiye (ethical code: 2011/034).

### Data availability

The data that support the findings of this study are available from the corresponding author upon reasonable request. 

### Author contributions

AÜ: running the project; ED: advising, supervising and managing the project; İÇ: advising and supervising the project.

### Conflict of interest

 The authors declare that they have no known competing financial interests or personal relationships that could have appeared to influence the work reported in this paper.

### Funding/support

The project was funded by the Scientific Research Projects Coordination Unit of Selçuk University, Konya, Türkiye for their financial support (Grant number: 11202027).

## References

[1] Velnar T, Bosnjak R, Garadisnik L (2022). Clinical applications of poly-methyl-methacrylate in neurosurgery: The in vivo cranial bone reconstruction. Journal of functional biomaterials.

[2] Durmus E, Celik I, Aydin MF, Yildirim G, Sur E (2008). Evaluation of the biocompatibility and osteoproductive activity of ostrich eggshell powder in experimentally induced calvarial defects in rabbits. Journal of biomedical materials research part B: applied biomaterials.

[3] Clavero J, Lundgren S (2003). Ramus or chin grafts for maxillary sinus inlay and local onlay augmentation: comparison of donor site morbidity and complications. Clinical implant dentistry and related research.

[4] Durmus E, Celik I, Ozturk A, Ozkan Y, Aydin MF (2003). Evaluation of the potential beneficial effects of ostrich eggshell combined with eggshell membranes in the healing of cranial defects in rabbits. Journalof international medical research.

[5] Fillingham Y, Jacobs J (2016). Bone grafts and their substitutes. Bone and joint journal.

[6] Valentini P, Abensur D (2003). Maxillary sinus grafting with anorganic bovine bone: a clinical report of long-term results. The international journal of oral and maxillofacial implants.

[7] Dupoirieux L, Pourquier D, Picot MC, Neves M Comparative study of three different membranes for guided bone regeneration of rat calvarial defects. International journal of oral and maxillofacial surgery 2001.

[8] Mann K, Siedler F (2004). Ostrich (Struthio camelus) eggshell matrix contains two different C-type lectin-like proteins. Isolation, amino acid sequence, and posttranslational modifications. Biochimica et biophysica acta.

[9] Dupoirieux L, Pourquier D, Picot MC, Neves M, Téot L (2001). Resorption kinetics of eggshell: an in vivo study. The journal of craniofacial surgery.

[10] Dupoirieux L, Pourquier D, Souyris F (1995). Powdered eggshell: a pilot study on a new bone substitute for use in maxillofacial surgery. Journal of cranio-maxillofacial surgery.

[11] Caliman LB, da Silva SN, Junkes JA, Della Sagrillo VP (2017). Ostrich eggshell as an alternative source of calcium ions for biomaterials synthesis. Materials research.

[12] Ghomi ER, Nourbakhsh N, Kenari MA, Zare M, Ramakrishna S (2021). Collagen-based biomaterials for biomedical applications. Journal of biomedical materials research Part B: applied biomaterials.

[13] Lee CH, Singla A, Lee Y (2001). Biomedical applications of collagen. International journal of pharmacology.

[14] Suppa P, Ruggeri A, Tay FR, Prati C, Biasotto M, Falconi M, Pashley DH, Breschi L (2006). Reduced antigenicity of type I collagen and proteoglycans in sclerotic dentin. The journal of dental research.

[15] Lynn AK, Yannas LV, Bonfield W (2004). Antigenicity and immunogenicity of collagen. Journal of biomedical materials research part B: applied biomaterials.

[16] Liu HW, Su WT, Liu Huang C, Huang CC (2022). Highly organized porous gelatin-based scaffold by microfluidic 3D-foaming technology and dynamic culture for cartilage tissue engineering. International journal of molecular sciences.

[17] Dubruel P, Unger R, Van Vlierberghe S, Cnudde V, Jacobs PJS, Schacht E, Kirkpatrick CJ (2007). Porous gelatin hydrogels: 2 In vitro cell interaction study. Biomacromoles.

[18] Taylor PM, Cass AEG, Yacoub MH (2006). Extracellular matrix scaffolds for tissue engineering heart valves. Progress in pediatric cardiology.

[19] Gorgieva S, Kokol V (2011). Collagen- vs Gelatine-Based Biomaterials and Their Biocompatibility: Review and Perspectives. Biomaterials applications for nanomedicine.

[20] Sohn DS, Moon JW, Moon KN, Cho SC, Kang PS (2010). New bone formation in the maxillary sinus using only absorbable gelatin sponges. The journal of oral and maxillofacial surgery.

[21] Lioubavina-Hack N, Karring T, Lynch SE, Lindhe J (2005). Methyl cellulose gel obstructed bone for mation by G BR: an experimental study in rats. Journal of clinical periodontology.

[22] Verna C, Bosch C, Dalstra M, Wikesjö UME, Trombelli L, Bosch C (2002). Healing patterns in calvarial bone defects following guided bone regeneration in rats A micro-CT scan analysis. Journal of clinical periodontology.

[23] Krithiga G, Sastryt P (2011). Preparation and characterization of a novel bone graft composite containing bone ash and egg shell powder. The bulletin of materials science.

[24] Redondo LM, Cantera JMG, Hernândez AV, Puerta CV (1995). Effect of particulate porous hydroxyapatite on osteoinduction of demineralized bone autografts in expeonmential reconstruction of the rat mandible. International journal of oral and maxillofacial surgery.

[25] Aaboe M, Pinholt ME, Hjorting-Hansen E (1995). Healing of experimentally created defects: a review. British journal of oral and maxillofacial surgery.

[26] Keipert S, Voigt R (1979). Interactions between macromolecular adjuvants and drugs Part 18: The binding behaviour of sodium carboxymethylcellulose and other macro-molecules towards streptomycin sulphfate. Pharmazie.

[27] Seto I, Asahina I, Oda M, Enomoto S (2001). Reconstruction of the primate mandible with a combination graft of recombinant human bone morpghogenetic protein-2 and bone marrow. Journal of oral and maxillofacial surgery.

[28] Ferreira JRM, Louro LHL, Costa AM, de Campos JB, Prado da Silva MH (2016). Ostrich eggshell as calcium source for the synthesis of hydroxyapatite, and hydroxyapatite partially substituted with zinc. Cerâmica.

[29] Park JW, Jang JH, Bae SR, An CH, Suh JY (2009). Bone formation with various bone graft substitutes in a critical-sized rat calvarial defect. Clinical oral implants research.

[30] Park JW, Bae SR, Suh JY, Lee DH, Kim SH, Kim H, Lee CS (2008). Evaluation of bone healing with eggshell-derived bone graft substitutes in rat calvaria: a pilot study. Journal of biomedical materials research part A.

[31] Luttikhuizen DT, Harmsen MC, Van Luyn MJA (2006). Cellular and molecular dynamics in the foreign body reaction. Tissue engineering.

[32] Rouahi M, Gallet O, Champion E, Dentzer J, Hardouin P, Anselme K (2006). Influence of hydroxyapatite microstructure on human bone cell response. Journal of biomedical materials research part A.

[33] Hench LL (1998). Biomaterials: a forecast for the future. Biomaterials.

[34] Hatano N, Shimizu Y, Ooya K (2004). A clinical long-term radiographic evaluation of graft height changes after maxillary sinus floor augmentation with 2:1 autogenous bone/xenograft mixture and simultaneous placement of dental implants. Clinical oral implants research.

